# NPY Released From GABA Neurons of the Dentate Gyrus Specially Reduces Contextual Fear Without Affecting Cued or Trace Fear

**DOI:** 10.3389/fnsyn.2021.635726

**Published:** 2021-05-26

**Authors:** Lucas B. Comeras, Noa Hörmer, Pradeepa Mohan Bethuraj, Ramon O. Tasan

**Affiliations:** Department of Pharmacology, Medical University Innsbruck, Innsbruck, Austria

**Keywords:** neuropeptide Y, NPY, fear, fear extinction, hippocampus, dentate gyrus

## Abstract

Disproportionate, maladapted, and generalized fear are essential hallmarks of posttraumatic stress disorder (PTSD), which develops upon severe trauma in a subset of exposed individuals. Among the brain areas that are processing fear memories, the hippocampal formation exerts a central role linking emotional-affective with cognitive aspects. In the hippocampus, neuronal excitability is constrained by multiple GABAergic interneurons with highly specialized functions and an extensive repertoire of co-released neuromodulators. Neuropeptide Y (NPY) is one of these co-transmitters that significantly affects hippocampal signaling, with ample evidence supporting its fundamental role in emotional, cognitive, and metabolic circuitries. Here we investigated the role of NPY in relation to GABA, both released from the same interneurons of the dorsal dentate gyrus (DG), in different aspects of fear conditioning. We demonstrated that activation of dentate GABA neurons specifically during fear recall reduced cue-related as well as trace-related freezing behavior, whereas inhibition of the same neurons had no significant effects. Interestingly, concomitant overexpression of NPY in these neurons did not further modify fear recall, neither under baseline conditions nor upon chemogenetic stimulation. However, potentially increased co-release of NPY substantially reduced contextual fear, promoted extinction learning, and long-term suppression of fear in a foreground context–conditioning paradigm. Importantly, NPY in the dorsal DG was not only expressed in somatostatin neurons, but also in parvalbumin-positive basket cells and axoaxonic cells, indicating intense feedback and feedforward modulation of hippocampal signaling and precise curtailing of neuronal engrams. Thus, these findings suggest that co-release of NPY from specific interneuron populations of the dorsal DG modifies dedicated aspects of hippocampal processing by sharpening the activation of neural engrams and the consecutive fear response. Since inappropriate and generalized fear is the major impediment in the treatment of PTSD patients, the dentate NPY system may be a suitable access point to ameliorate PTSD symptoms and improve the inherent disease course.

## Introduction

Neuropeptide Y (NPY) is a 36 amino acid peptide neurotransmitter (Tatemoto et al., 1982) and extensively expressed in the peripheral and central nervous systems (Gehlert et al., 1987; Morris, 1989). By acting on at least five different Gi/o-coupled receptors, it modulates multiple physiological processes, such as energy homeostasis and emotional-affective behaviors (Hokfelt et al., 2007; Gilpin, 2012; Loh et al., 2015; Reichmann and Holzer, 2016; Schmeltzer et al., 2016; Tasan et al., 2016). Endogenous NPY has been suggested as a resilience factor associated with post-traumatic stress disorder (PTSD); however, the underlying mechanisms are still unclear (Kautz et al., 2017).

A hallmark of PTSD is dysregulated fear, which often appears as fear generalization and the inability to suppress and extinguish established fear memories. Individual aspects of PTSD can be modeled by Pavlovian fear conditioning, a simple form of associative learning, in which an initially neutral stimulus (CS, conditioned stimulus) or a specific context is repetitively paired with an aversive signal, for instance, an electric foot shock (US, unconditioned stimulus; LeDoux, 2000). Multiple brain areas, including the amygdala, hippocampus and, cortical structures, are controlling fear learning and consecutive adaptive behaviors (Ehrlich et al., 2009; Pape and Pare, 2010; Bowers et al., 2012; Tasan et al., 2016; Comeras et al., 2019). The hippocampal formation, for instance, is processing and integrating particular contextual contents about place and time into other fear-relevant brain circuitries (Maren and Fanselow, 1995; Cox et al., 2013; Hubner et al., 2014; McDonald and Mott, 2017). These phenomena can be investigated by cued, contextual and trace fear conditioning.

Synaptic transmission in the hippocampus is generally organized in an excitatory tri-synaptic pathway (Amaral and Witter, 1989) with a multitude of highly specialized inhibitory interneurons generating appropriate fine-tuning by the release of GABA and several neuromodulators. NPY is one of these neuromodulators and in the hippocampus, it can activate postsynaptic Y1 and presynaptic Y2 receptors (Stanic et al., 2011). NPY is usually co-released with GABA from various types of interneurons, while Y1 and Y2Rs are both expressed predominantly on glutamatergic projection neurons (Kopp et al., 2002; Stanic et al., 2006, 2011), such as CA pyramidal neurons and dentate granule cells. Ample evidence emphasizes strong fear-reducing properties of exogenously applied NPY in various rodent models (Flood et al., 1987, 1989; Gutman et al., 2008; Fendt et al., 2009; Tasan et al., 2016). In particular, Y1Rs seem to suppress fear learning in multiple brain areas, including the amygdala and hippocampus (Gutman et al., 2008; Lach and De Lima, 2013). On the other hand, activation of Y2Rs also inhibits acquisition and promotes extinction of fear memories (Verma et al., 2012), predominantly in the extended amygdala and hippocampus (Verma et al., 2015, 2018a,b; Tasan et al., 2016; Hormer et al., 2018). For example, local re-expression of hippocampal Y2Rs in Y2KO mice resulted in specific suppression of fear, by reducing fear recall and promoting fear extinction (Hormer et al., 2018).

Thus, while several pharmacological and genetic studies indicate a role of exogenous NPY in the modulation of fear memories, a direct investigation of the specific contribution of endogenous NPY specifically co-released from GABAergic interneurons would be of particular interest.

Here, we focused on the contribution of local inhibitory neurons to hippocampus-dependent fear processing and investigated the specific role of the co-released neuromodulator NPY. We activated and inhibited dorsal DG GABA neurons during cued and trace fear recall. Furthermore, concomitant overexpression of NPY in activated GABA neurons was used to identify the specific role of co-released NPY in various aspects of fear conditioning, including cued, trace, and contextual fear. To narrow down the underlying neuronal subpopulations, we analyzed the co-localization of NPY with specific neurochemical markers in different sub-regions of the dorsal and ventral hippocampus. Our data suggest that the co-release of NPY from hippocampal GABA neurons modulates highly selective aspects of hippocampal fear conditioning and extinction learning.

## Materials and Methods

### Animals

Adult male VGAT-cre (B6.FVB-Tg(Slc32a1-cre)2.1Hzo/FrkJ, The Jackson Laboratory, USA) mice, NPY-GFP (B6.FVB-Tg(Npy-hrGFP)1Lowl/J, The Jackson Laboratory, USA) mice and wild type mice (WT, C57BL/6NCrl, Charles River Laboratories, Sulzfeld, Germany) were bred in the Department of Pharmacology at the Medical University Innsbruck, Austria. Following weaning they were group-housed in groups of three to five animals per cage under standard laboratory conditions (12 h light/dark cycle, lights on at 07:00). Both transgenic mouse lines were backcrossed with C57BL/6NCrl (Charles River Laboratories, Sulzfeld, Germany) for at least 10 generations.

All procedures involving animals and animal care were conducted in accordance with international laws and policies (Directive 2010/63/EU of the European Parliament and of the council of 22 September 2010 on the protection of animals used for scientific purposes; Guide for the Care and Use of Laboratory Animals, U.S. National Research Council, 2011, and ARRIVE (Animal Research: Reporting *in vivo* Experiments) guidelines) and were approved by the Austrian Ministry of Science. All effort was taken to minimize the number of animals used and their suffering.

### Viral Vectors

Plasmids for recombinant adeno-associated viral (rAAV) vectors, rAAV6-hSyn-DIO-hM3D(Gq)-mCherry (Addgene 44361, B. Roth) and rAAV6-hSyn-DIO-hM4D(Gi)-mCherry (Addgene 44362, B. Roth), were kindly provided by B. Roth, obtained from Addgene (USA) and packaged in our laboratory as described in detail previously (Tasan et al., 2010; Verma et al., 2015). A codon-optimized plasmid containing the coding sequence of mouse preproNPY (NCBI Reference Sequence: NM_023456.3) was ordered from GeneScript (USA) and cloned into the open-reading frame of Addgene plasmid 26968 (rAAV6-EF1a-DIO-preproNPY). In order to promote the proper processing of transgene preproNPY into functional NPY, we did not include an additional reporter gene into the final construct. All vectors were diluted to obtain final titers of 10^12^ gp/ml.

### Stereotaxic Microinjections

Mice were anesthetized (150 mg/kg ketamine ip, Ketasol, Animedica) and received analgesia (1.5 mg/kg Meloxicam s.c., Movalis^®^, Boehringer Ingelheim, Germany) prior to mounting in a stereotaxic frame (David Kopf Instruments, USA). During the procedure, anesthesia was maintained by 1–3% sevoflurane (Sevorane, AbbVie) inhalation. Mice were bilaterally injected with 0.5 μl of rAAV into the dorsal DG (coordinates relative to bregma in mm: dorsoventral −2.0, rostrocaudal −2.0, mediolateral ±1.5) by micropumps (Nexus 3000, Science Products GmbH, Germany) at a flow rate of 0.1 μl/min. The injection site was verified by immunohistochemistry for RFP/mCherry requiring that >90% of labeled neuronal cell bodies must reside within the dorsal DG, excluding, in particular, the ventral DG and other parts of the hippocampal formation, such as CA1 to CA3 areas.

### Fear Conditioning

Several groups of VGAT-cre mice were compared in behavioral experiments. For manipulating DG GABA neurons, rAAV6-hSyn-DIO-hM3D(Gq)-mCherry and rAAV6-hSyn-DIO-hM4D(Gi)-mCherry vectors were locally injected into the dorsal DG to activate or inhibit DG GABA neurons, respectively by intraperitoneal (ip) injection of clozapine-N-oxide (CNO, ip, 1 mg/kg, Carbosynth, UK) 30 min before behavioral testing (Gq and Gi groups). Pilot studies revealed that a dose of 1 mg/kg of CNO was sufficient to activate and inhibit hM3D (Gq) and hM4D (Gi)-expressing neurons, respectively. As controls, both groups, Gq and Gi, were injected ip with 0.9% NaCl before testing (saline group). To investigate the role of concomitantly released NPY, a group of mice was injected with a combination of rAAV6-hSyn-DIO-hM3D (Gq)-mCherry and rAAV6-EF1a-DIO-preproNPY (Gq + NPY group) into the dorsal DG and compared to Gq littermates (Gq group), both injected ip with CNO before the respective behavioral test.

For behavioral testing, mice were single-housed in cages covered by filter tops with food and water available *ad libitum* and transferred to the experimental rooms 3 days before behavioral testing. Fear conditioning experiments were performed as described previously (Verma et al., 2012; Hormer et al., 2018).

In brief, trace fear acquisition was performed in context A, consisting of a fear conditioning arena made of a transparent acrylic chamber with a metal grid floor (Ugo Basile, Italy). Illumination was 80 lux and the chambers were cleaned with 70% ethanol. On acquisition day, after a 120 s habitation period, 5 CS (white noise, 20 s, 70 db) each followed by a 5 s trace period and a US (0.5 mA foot-shock for 2 s) were provided with a random inter-trial interval of 40–60 s. For testing of contextual fear, mice were placed in context A for 25 min on day 2. On the third day, to test for CS-induced and trace-induced fear expression the mice received 25 CS separated by 20 s pauses in context B, consisting of an arena with black and white striped walls, white floor, 10 lux illumination and an odor of 1% acetic acid. The first 5 CS were used for testing fear recall and the next 20 CS as cued fear extinction. The 5 s period immediately following each CS was used for analysis of trace fear.

Contextual foreground conditioning was performed in context A. Following a habituation period of 120 s, mice were fear conditioned to the context by delivery of 5 US (unconditioned stimulus, 0.5 mA foot-shock for 2 s) with a random inter-trial interval of 40–60 s. Freezing behavior was determined in the 20 s preceding each US (PreUS). After this procedure, mice remained in the test apparatus for an additional 120 s and were then returned to their home cage. On the next day, fear test and fear extinction were performed by exposing the mice to the same context A for 25 min. The first 5 min were considered as fear recall and the next 20 min as fear extinction. Finally, recall of context extinction was tested on the next day by putting the mice into the same context for 5 min. For re-conditioning experiments, mice were exposed to two additional context fear extinction sessions (each 25 min) on days 4 and 5, followed by 1 week of resting in their home cage. Re-conditioning and CNO injection before CS/trace fear recall was performed similar to trace fear conditioning described above but in a different context. Freezing behavior was recorded and quantified by a pixel based analysis software and expressed as percentage freezing per CS, trace or min for cued, trace, and context fear, respectively (AnyMaze, Stoelting, USA). The parameters of the analysis software were validated by manual measurements of time spent freezing, defined as the absence of all movements except those necessary for breathing.

### Feeding Drinking and Activity

Food intake, water intake, and home-cage activity were recorded continuously using an automated home cage phenotyping system (PhenoMaster, TSE, Bad Homburg, Germany). Mice had access *ad libitum* to food and water and they were kept under standard laboratory conditions (12 h light/dark cycle, lights on at 07:00).

### Immunohistochemistry

Immunohistochemistry was performed as described earlier (Tasan et al., 2010, 2011; Wood et al., 2016). Mice were injected ip with thiopental (150 mg/kg, ip, Sandoz, Austria) for deep anesthesia. Transaortal perfusion with phosphate buffered saline (PBS) at room temperature for 3 min followed by 4% paraformaldehyde (PFA) at 4°C for 10 min was performed by a peristaltic pump at a flow rate of 9 ml/min (Ismatec, IPC, Cole-Parmer GmbH). Subsequently, brains were removed and postfixed in 4% PFA for 90 min at 4°C, cryoprotected for 48 h in 20% sucrose at 4°C and then snap-frozen in isopentane (2-methylbutane, Merck KGaA, Germany) for 3 min at −60°C. Brains were transferred to pre-cooled open tubes and stored at −70°C until further use.

For immunohistochemistry, coronal 40 μm sections were cut on a cryostat from rostral to caudal and collected in Tris-buffered saline (TBS) + 0.1% sodium azide. Sections from the hippocampus were incubated for 30 min in TBS-triton (0.4%) and 90 min in 10% normal horse serum. Subsequently, sections were incubated overnight with the first primary antibody (Table 1) diluted in 10% serum containing 0.1% sodium azide. After washing with TBS-buffer 3 × 5 min, the secondary antibody was added to the sections for 150 min. Then, sections were incubated in the dark for 8 min in TSA-TAMRA (in-house, 1:2,000) staining solution. For dual labeling immunohistochemistry, sections were incubated in 100 mM sodium azide for 45 min. Subsequently, they were rinsed with TBS-buffer 3 × 5 min and incubated with the corresponding second primary antibody (diluted in 10% serum containing 0.1% sodium azide) overnight (Table 1). On the next day, after washing with TBS-buffer 3 × 5 min, TSA biotin was added to the sections for 6 min, followed by rinsing 3 × 5 min in TBS buffer and then incubated for 100 min in a solution of streptavidin dylight 649 (Vector Laboratories, Inc., USA) in TBS buffer. Fluorescently stained sections were mounted on slides using gelatine and cover-slipped with glycerol-DABCO anti-fading mounting medium.

**Table 1:** Primary and secondary antibodies.

	Species	Code	Source	Dilution
**Primary antibodies**
c-Fos	Rabbit	226003	Synaptic systems	1:10,000
GABA	Rabbit	A2052	Sigma	1:2,000
NPY	Rabbit	In-house	Bellmann et al. (1991)	1:10,000
Parvalbumin	Rabbit	PV 25	Swant	1:20,000
RFP	Rabbit	600-401-379	Rockland	1:10,000
Somatostatin	Rabbit	In-house	Sperk and Widmann, 1985	1:20,000
**Secondary antibodies**
Anti-Rabbit-HRP	Goat	P0448	Dako	1:250
Anti-Rabbit-HRP	Donkey	A16023	Invitrogen	1:500
**HRP-subtrate**				
Streptavidin 649			Vector Laboratories	1:100
TSA TAMRA			Thermo Fisher	1:2,000
TSA Biotin			Thermo Fisher	1:100

### Imaging and Counting

Images were taken using a fluorescent microscope (Zeiss Axio Imager M1, Germany) equipped with a halogen light source, respective filter sets, a Hamamatsu monochrome camera (Hamamatsu ORCA ER C4742-80-12AG, Japan), and Improvision Openlab software (PerkinElmer, USA). For each mouse, numbers of immuno-positive neurons per sub-region were obtained bilaterally from coronal sections comprising the dorsal (bregma −1.70 to −2.30 mm) and ventral hippocampus (bregma −2.92 to −3.16 mm; Paxinos and Franklin, 2004). In order to assess the overexpression of NPY in the DG, mean gray values of the DG were obtained using ImageJ FIJI (ImageJ, USA) from inverted photomicrographs of NPY immunohistochemistry of the dorsal hippocampus. Mean gray values were calculated using a circular area placed in the center of each DG. The mean gray value of the background (area of the slide without positive signal) was calculated for each slide and subtracted from the mean gray value of the DG. Relative optical density (ROD) was calculated using the formula: ROD = log (255/mean gray value).

### Statistical Analysis

Data are shown as means ± SEM and were analyzed for normal distribution and equal variances using GraphPad Prism software (Prism 8, GraphPad Software Inc.). Measurements of treatment, time, and interaction were analyzed by repeated two-way ANOVA, followed by Tukey’s *post hoc* test. Comparisons of the two groups were done by unpaired or paired Student’s *t*-tests or Fisher’s exact test for proportions.

## Results

### Activation of GABAergic Neurons of the Dentate Gyrus Reduces Trace Fear Recall

**Figure 1:**
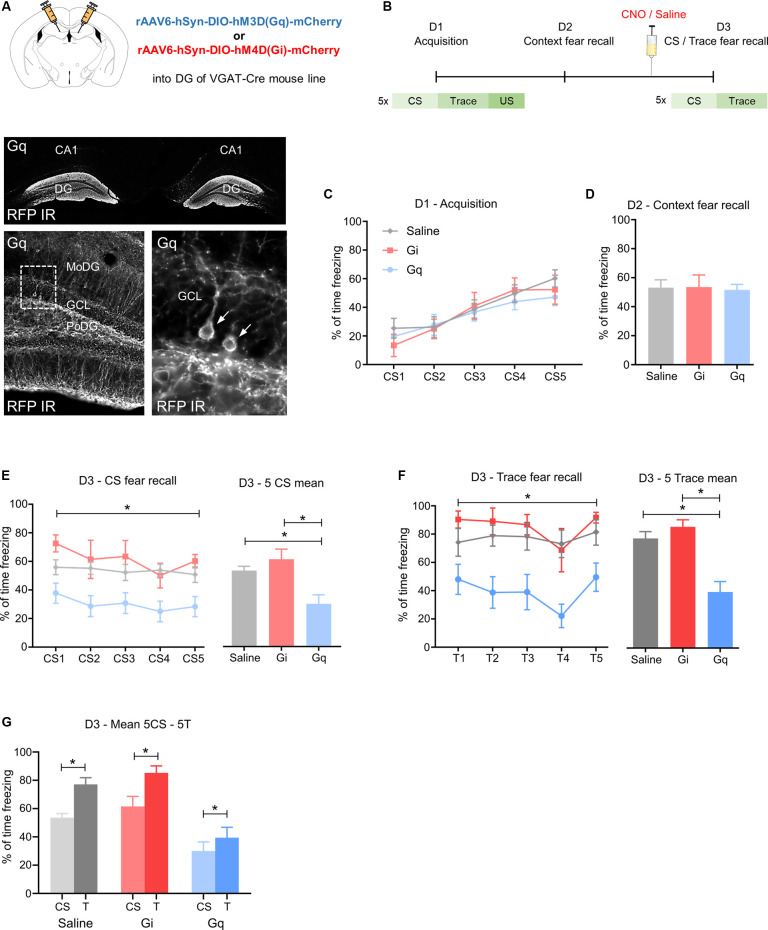
Activation of GABA neurons in the dorsal dentate gyrus reduces fear expression. **(A)** Upper image: schematic illustration of the injection site of rAAV-hSyn-DIO-hM3DGq-mCherry (Gq) or rAAV-hSyn-DIO-hM4DGi-mCherry (Gi) in the dorsal dentate gyrus (DG) of VGAT-cre mice. Lower image: immunohistochemical distribution of mCherry in the dorsal DG (arrows point towards individual immuno-positive GABA neurons). **(B)** Schematic fear conditioning protocol. On day 1 (D1), five pairings of a CS (20 s) followed by a 5 s trace and a 0.5 mA footshock were delivered with a variable inter-trial interval of 40–60 s. On day 2 (D2), mice were placed into the acquisition context for 25 min. On day 3 (D3), mice were injected with 1 mg/kg clozapine-N-oxide (CNO) 30 min before fear recall testing, consisting of 5CS presentations in a different context. **(C)** Equal fear acquisition for all groups, **(D)** no change in contextual fear, **(E)** during testing of CS-induced fear recall, Gq group showed a lower percentage of freezing than Gi and saline during 5 CS as demonstrated by two-way ANOVA for repeated measurements and Tukey’s *post hoc* test. **(F)** A similar reduction of the percentage of freezing was seen during the trace period. **(G)** Saline, Gq, and Gi injected mice showed a higher percentage of freezing during the trace period compared to the CS, suggesting intact stimulus contingency. Data are presented as means ± SEM, *P < 0.05. Saline: *n* = 11, Gi: *n* = 6, Gq: *n* = 14. Abbreviation: CS, conditioned stimulus.

To investigate the role of hippocampal GABA neurons in fear processing we injected male VGAT-cre mice with an activatory (rAAV-hSyn-DIO-hM3DGq) or inhibitory (rAAV-hSyn-DIO-hM4DGi) DREADD vector (Figure 1A) into the dorsal DG. A detailed analysis of the injection site revealed that transgene expression was confined to the dorsal DG, but absent from its ventral parts (Supplementary Figure 1A). Two weeks after rAAV injection, hippocampus-dependent fear processing was tested by trace fear conditioning, a paradigm that relies on dorsal hippocampal signaling (Misane et al., 2005; Esclassan et al., 2009). Trace fear conditioning was optimized in pilot studies for C57Bl6N/Crl mice. Using a 5 s trace interval provided optimal conditions for differential freezing behavior. Thus, the highest freezing levels were detected during the trace period compared to medium and lowest freezing behavior during CS and inter-trial intervals, respectively (Supplementary Figure 2B). Following fear acquisition and contextual testing, hippocampal interneurons were activated or inhibited by peripheral injection of CNO or saline in controls 30 min before fear recall (Figure 1B).

In the absence of CNO injection, Gi, Gq, and saline control groups displayed equal fear acquisition (Figure 1C) and context fear expression (Figure 1D). However, on day 3, when CNO or saline was injected 30 min before fear testing, CS-induced and trace-induced freezing was reduced in Gq mice, as demonstrated by repeated two-way ANOVA (Figure 1E, *F*_(2,28)_ = 7.998, *P* = 0.0018 for treatment). This difference was mainly attributed to a reduction in CS-induced freezing of the Gq mice injected with CNO compared to Gi and saline groups (Figure 1E saline vs. Gq: 23.5, 95%—CI (4.93, 42.07), *P* = 0.0109; Gi vs. Gq: 31.4, 95%—CI (8.92, 53.89), *P* = 0.0049), indicating reduced fear expression upon activation of DG GABA neurons. Furthermore, during fear testing, Gq mice injected with CNO also exhibited lower freezing during the initial five traces when compared to saline-injected controls and to the CNO injected Gi group (Figure 1F; *F*_(2,28)_ = 14.04, *P* = 0.0001 for treatment). Interestingly, the contingency of CS and trace was not influenced by treatment in any of the groups as demonstrated by the consistently increased freezing levels to the trace when compared to the preceding CS-period (Figure 1G; Gq: *t*_(14)_ = 2.573, *P* = 0.0232, Gi: *t*_(6)_ = 4.766, *P* = 0.0050, saline: *t*_(11)_ = 4.964, *P* = 0.0006). Together these data suggest that the activation of GABA neurons in the dorsal DG reduces CS-related and trace-related fear while keeping the identification of salient predictive stimuli unaffected. Analyses of the injected brain areas with dual immunohistochemistry revealed RFP expression in somatostatin (SST)- and parvalbumin (PV)-expressing interneurons of the dorsal DG (Supplementary Figures 1B,C).

### Overexpression of NPY in GABAergic Neurons of the Dorsal Dentate Gyrus Does Not Affect Trace Fear Recall

**Figure 2:**
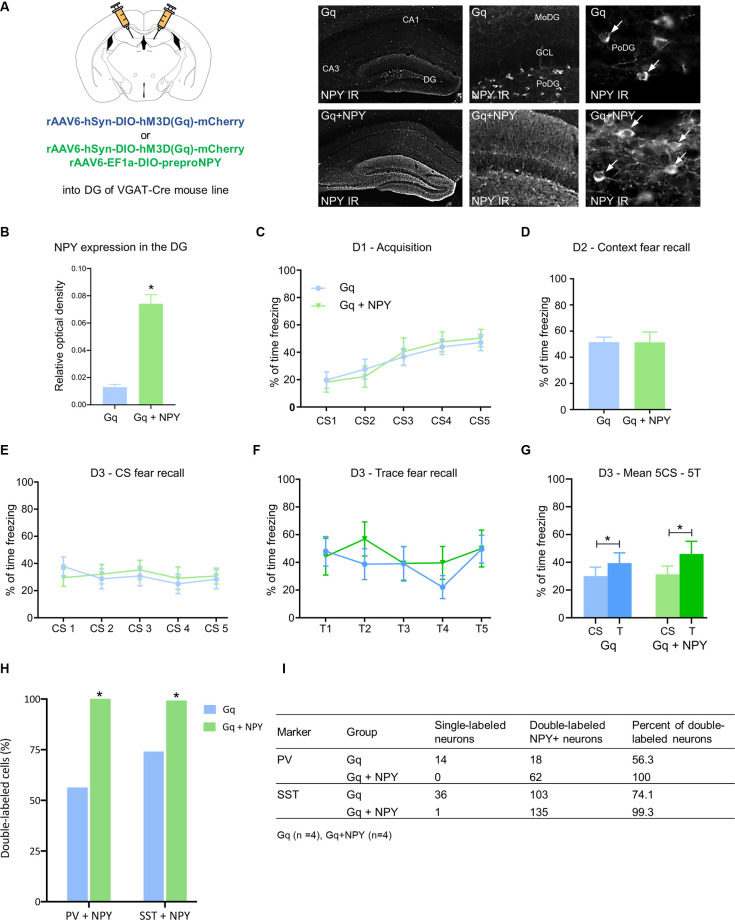
Overexpression of neuropeptide Y (NPY) in dentate gyrus GABA neurons does not further reduce CS-related and trace-related fear expression upon chemogenetic activation. **(A)** Photomicrographs of NPY immunohistochemistry in coronal mouse brain sections and quantification of relative optical density (ROD) demonstrate higher expression of NPY in the dorsal hippocampus of mice injected with rAAV-hSyn-DIO-hM3DGq-mCherry/rAAV-EF1a-DIO-NPY (Gq + NPY) than those injected with rAAV-hSyn-DIO-hM3DGq-mCherry (Gq) alone. **(B)** Increased expression of NPY-IR in the dorsal DG of a Gq + NPY injected mice compared to Gq injected controls. **(C)** No change in percentage of freezing during fear acquisition on day 1 (D1) and **(D)** equal freezing during contextual fear testing on day 2 (D2). **(E)** Similarly, there was no difference in freezing time during the CS and **(F)** trace period on day 3 (D3) following injection of CNO to activate dentate GABA neurons. **(G)** Both, Gq and Gq + NPY injected mice showed higher freezing during trace than during CS-period. **(H)** Quantification of dual-immunofluorescence demonstrating increased expression of NPY-IR in parvalbumin (PV) and somatostatin (SST) neurons of the dorsal DG of Gq + NPY mice compared to Gq-injected controls. **(I)** Tabular results for individual groups of DG interneurons in Gq + NPY and Gq mice. Data are presented as means ± SEM, *P < 0.05. Gq: *n* = 14, Gq + NPY: *n* = 11.

Under physiological conditions, NPY in the hippocampus is almost exclusively expressed and released from specific populations of GABAergic interneurons. Consequently, to explore the role of NPY in hippocampus-dependent fear processing, we injected a combination of rAAV-hSyn-DIO-hM3DGq and rAAV-EF1a-DIO-NPY into the dorsal DG of VGAT-cre mice (Figure 2A). This approach resulted in an overexpression of NPY specifically in DG GABA neurons (Figure 2B; *t*_(8)_ = 8.324, *P* = 0.0000085) and provided the opportunity to induce locally restricted NPY release together with GABA by peripheral injection of CNO.

No significant changes in freezing time were observed during fear acquisition (Figure 2C) and context fear testing (Figure 2D) under baseline conditions, suggesting that the isolated overexpression of NPY in DG GABA neurons does not modify fear learning. Similarly, when CNO was injected before cued CS-induced and trace-induced fear tests, Gq + NPY mice displayed equal freezing to the CS (Figure 2E) and trace (Figure 2F) as Gq mice. Importantly, the contingency of CS and trace was maintained in both groups (Figure 2G; Gq: *t*_(14)_ = 2.573, *P* = 0.0232, Gq + NPY: *t*_(11)_ = 2.903, *P* = 0.0158). These data indicate that NPY released from hippocampal GABA neurons does not interfere with CS-related nor trace-related fear recall. Analyses of the injected brain areas with dual immunohistochemistry revealed NPY expression in almost all PV- and SST-expressing interneurons of the dorsal DG (Figures 2H,I).

### Overexpression of NPY in GABAergic Neurons of the Dorsal Dentate Gyrus Reduces Context Fear Recall

**Figure 3:**
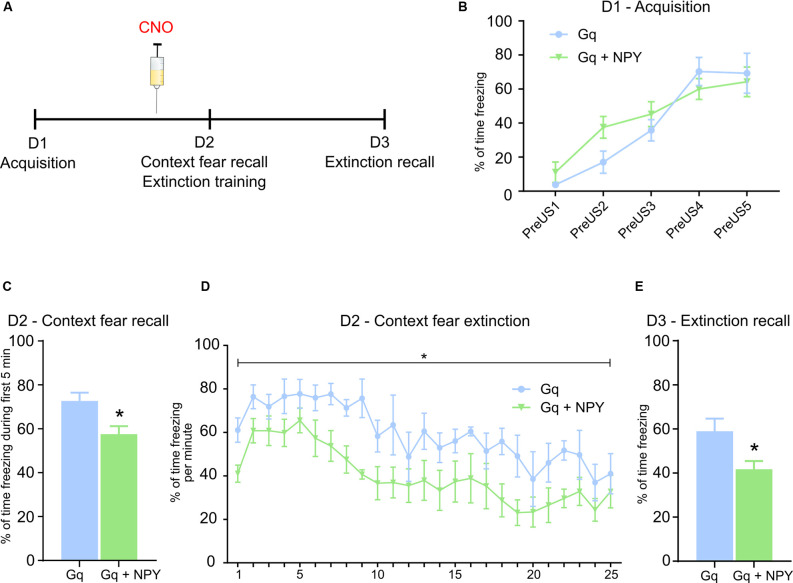
NPY over-expression in GABAergic neurons of the dorsal dentate gyrus reduces context fear expression upon activation. **(A)** Foreground context fear protocol: On day 1 (D1), during fear acquisition mice received 5 US separated by a variable time interval. On day 2 (D2), mice were injected 30 min before the test with CNO (ip, 1 mg/kg). Fear testing and extinction training: On day 3 (D3), mice were placed again into the same context for 5 min to test for extinction recall in the absence of CNO injection. **(B)** No change in percentage of freezing measured during the 20 s period before each US (PreUS1–5) between Gq and Gq + NPY injected mice. **(C)** During testing of contextual fear recall following peripheral injection of CNO, Gq + NPY mice exhibited a lower percentage of freezing compared to Gq mice. **(D)** Similarly, Gq + NPY mice displayed a lower percentage of freezing during context fear extinction upon peripheral injection of CNO. **(E)** On D3, in the absence of peripheral CNO injection, Gq + NPY mice showed a lower percentage of freezing during 5 min of context fear recall. Data are presented as means ± SEM, *P < 0.05. Gq: *n* = 4, Gq + NPY: *n* = 5. Abbreviation: US, unconditioned stimulus.

To investigate whether NPY released from DG GABA neurons has a role in specific forms of Pavlovian fear conditioning, such as foreground contextual fear, we subjected Gq and Gq + NPY injected mice to pure context fear conditioning (Figure 3A). Overexpression of NPY did not alter fear acquisition (Figure 3B) in the absence of CNO. However, 24 h after acquisition, context fear recall was performed in the presence of CNO (Figures 3A–C), followed by extinction learning (Figures 3A–D). Compared to Gq mice, Gq + NPY injected mice showed lower levels of freezing in contextual fear recall on day 2, performed 30 min after injection of CNO (Figure 3C; *t*_(7)_ = 2.896, *P* = 0.0231). Similarly, Gq + NPY injected mice also showed an accelerated extinction learning in the presence of CNO-mediated activation of DG GABA neurons (Figure 3D, *F*_(1,7)_ = 10.88, *P* = 0.0131 for treatment). Extinction recall was performed on day 3 without CNO injection (Figure 3E). Importantly, contextual freezing was also reduced during extinction recall in Gq + NPY mice compared to Gq alone (Figure 3E; *t*_(7)_ = 2.604, *P* = 0.0352). These data indicate that NPY reduces the expression and promotes extinction especially of foreground context fear when released from GABAergic interneurons of the dorsal DG. To substantiate these findings, we subjected the same mice to an extensive context extinction protocol and re-conditioned them in the same trace fear paradigm as described above. Importantly, there was no change in CS or trace fear recall upon injection of CNO (Supplementary Figures 3A–C), confirming the previous findings (Figures 2E–G) and strengthening the selectivity of NPY for foreground contextual fear (Figures 3C–E). Interestingly, context fear and trace fear expression were not correlated in these mice (Supplementary Figure 3C).

### Overexpression of NPY Did Not Change Food Intake, Water Intake or Activity

**Figure 4:**
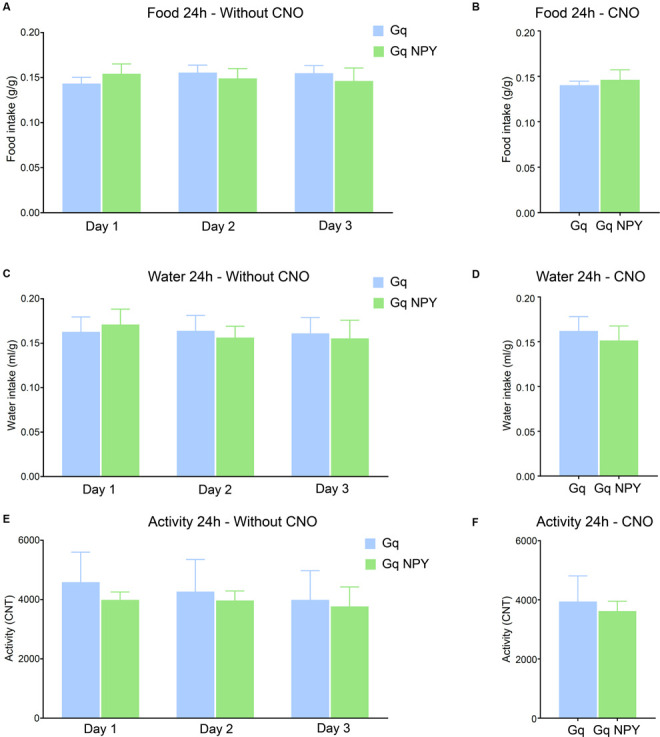
Activation of GABAergic neurons in the dorsal dentate gyrus with or without overexpression of NPY does not alter food intake, water intake, or home cage activity. **(A)** No difference in cumulative 24 h food intake per body weight (g/g) across three consecutive light/dark cycles. **(B)** No change in cumulative 24 h food intake per body weight after ip injection of CNO. **(C)** No difference in cumulative 24 h water intake per body weight (ml/g) on three consecutive days. **(D)** No change in cumulative 24 h water intake per body weight after ip CNO injection. **(E)** There was also no difference in cumulative 24 h home cage activity under baseline conditions or **(F)** after peripheral CNO injection. Gq: *n* = 4, Gq + NPY: *n* = 4.

Ample evidence supports an important role of NPY in the modulation of food intake and metabolic processes, in particular when released from neurons of the arcuate hypothalamic nucleus. In order to identify if also hippocampal NPY plays a role in metabolic processes, we measured food intake, water intake and home-cage activity in Gq and Gq + NPY mice, both in the absence and presence of ip CNO injections. Food and water intake and general home-cage activity was neither changed by DG NPY overexpression (Figures 4A–E) nor during concomitant activation of DG GABA neurons by ip CNO injection (Figures 4B–F). These data exclude the involvement of unspecific alterations in motor activity and confirms that NPY in the dorsal DG does not affect metabolically induced behaviors.

### Distribution of NPY Neurons in the Different Hippocampal Subregions

**Figure 5:**
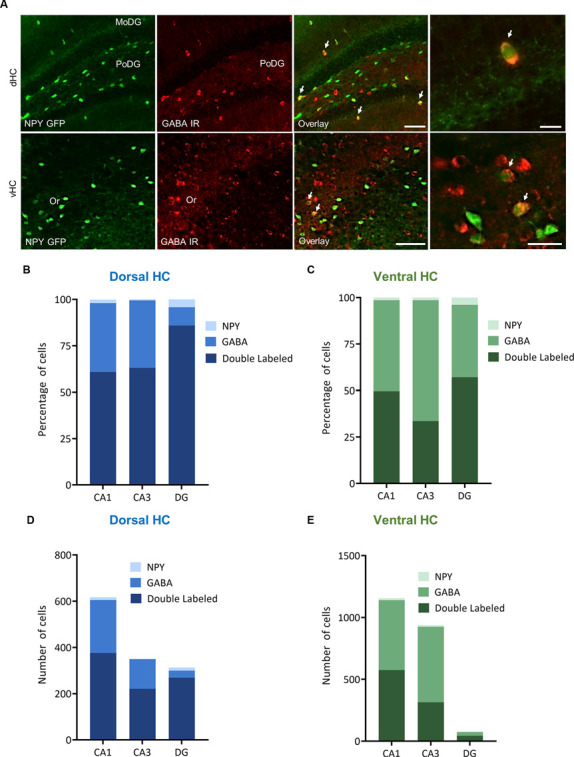
Distribution and quantification of co-expression of GABA with NPY in the dorsal (dHC) and ventral (vHC) mouse hippocampus. **(A)** Dual immunohistochemistry for GABA and NPY-GFP auto-fluorescence in the dorsal (dHC) and ventral hippocampus (vHC) of NPY-GFP mice. Cells in green indicate NPY-GFP + neurons and cells in red indicate GABA expressing neurons. **(B)** Histograms showing the percentage of NPY-GFP neurons in CA1, CA3, and DG of the dorsal hippocampus that are co-labeling with GABA. **(C)** Histograms showing the percentage of NPY-GFP neurons in CA1, CA3, and DG of the ventral hippocampus that are co-labeling with GABA. Note that NPY neurons are exclusively GABAergic. **(D)** Number of NPY-GFP and GABA neurons in CA1, CA3, and DG of the dorsal and **(E)** ventral hippocampus. Scale bars: lower magnification 200 μm (third column), higher magnification 50 μm (last column). Abbreviations: MoDG, molecular layer of the dentate gyrus; PoDG, hilus/polymorph layer of the dentate gyrus; Or, stratum oriens.

**Figure 6:**
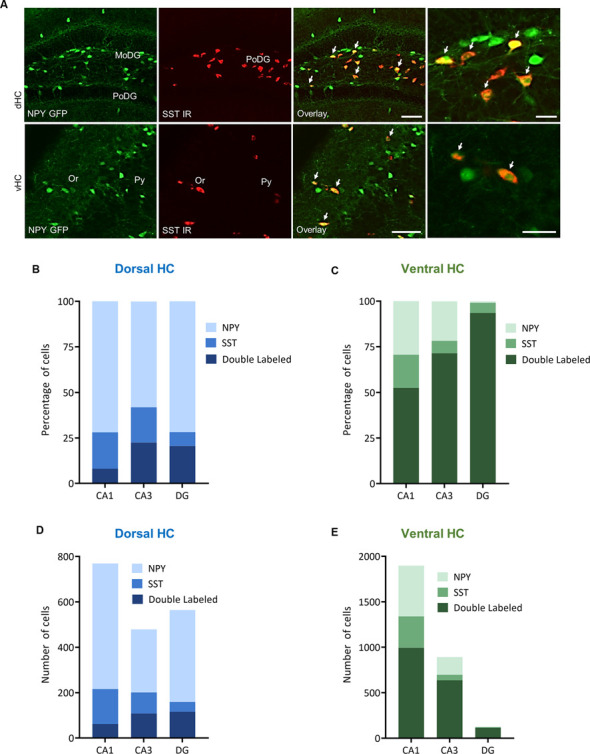
Distribution and quantification of co-expression of somatostatin (SST) with NPY in the dorsal (dHC) and ventral (vHC) mouse hippocampus. **(A)** Dual immunohistochemistry for SST and NPY-GFP auto-fluorescence in the dorsal (dHC) and ventral hippocampus (vHC) of NPY-GFP mice. Cells in green indicate NPY-GFP + neurons and cells in red indicate SST expressing neurons. **(B)** Histograms showing the percentage of NPY-GFP neurons in CA1, CA3, and DG of the dorsal hippocampus that are co-labeling with SST. **(C)** Histograms showing the percentage of NPY-GFP neurons in CA1, CA3, and DG of the ventral hippocampus that are co-labeling with SST. In the ventral hippocampus a very high proportion of NPY neurons co-express SST. **(D)** Number of NPY-GFP and SST neurons in CA1, CA3, and DG of the dorsal and **(E)** ventral hippocampus. Scale bars: lower magnification 200 μm (third column), higher magnification 50 μm (last column). Abbreviations: MoDG, molecular layer of the dentate gyrus; PoDG, hilus/polymorph layer of the dentate gyrus; SGZ, subgranular zone; Or, stratum oriens; Py, stratum pyramidale.

**Figure 7:**
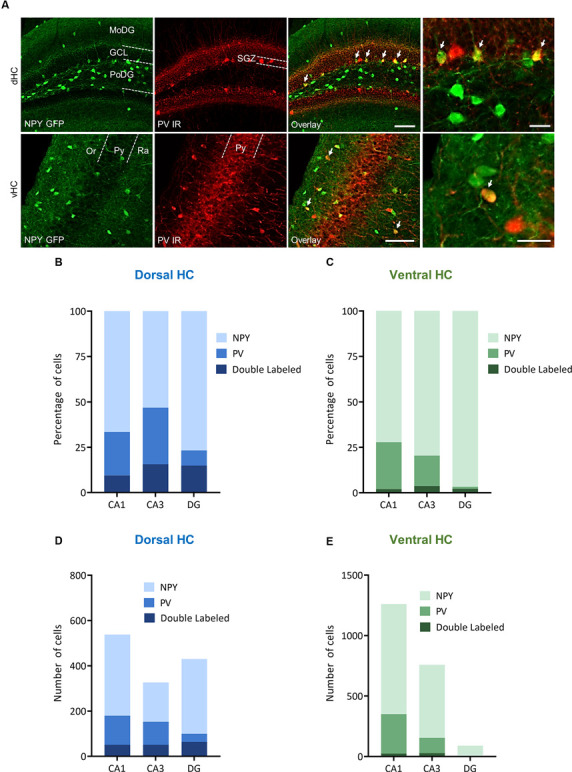
Distribution and quantification of co-expression of parvalbumin (PV) with NPY in the dorsal (dHC) and ventral (vHC) mouse hippocampus. **(A)** Immunohistochemical labeling for PV and NPY-GFP auto-fluorescence depicting dual-labeled neurons (upper panel: dorsal dentate gyrus, lower panel: ventral hippocampus CA1, arrows depict dual-labeled neurons) of NPY-GFP mice. Cells in green indicate NPY-GFP + neurons and cells in red indicate PV expressing neurons. **(B)** Histograms showing the percentage of NPY-GFP neurons in CA1, CA3, and DG of the dorsal hippocampus that are co-labeling with PV. **(C)** Histograms showing the percentage of NPY-GFP neurons in CA1, CA3, and DG of the ventral hippocampus that are co-labeling with PV. In the ventral hippocampus the amount of NPY and PV dual labeling is very low. **(D)** Number of NPY-GFP and PV neurons in CA1, CA3, and DG of the dorsal and **(E)** ventral hippocampus. Scale bars: lower magnification 200 μm (third column), higher magnification 50 μm (last column). Abbreviations: MoDG, Molecular layer of the dentate gyrus; GCL, granule cell layer; PoDG, hilus/polymorph layer of the dentate gyrus; SGZ, subgranular zone; Or, stratum oriens; Py, stratum pyramidale; and Ra, stratum radiatum.

In order to specify the underlying neuronal subpopulations that may be responsible for NPY-mediated reduction of contextual fear, we performed immunohistochemical analyses in NPY-GFP mice. In the hippocampus, NPY was almost exclusively expressed in GABA-expressing neurons (Figures 5A–E; Table 2). According to their projection targets, gene expression profile, and electrophysiological properties, inhibitory interneurons in the hippocampus are highly specialized and diverse. Here, we showed that NPY was expressed mainly by SST neurons. In the dorsal hippocampus, the percentage of NPY and SST co-expressing neurons was 8.1, 22.5, and 20.6% for the CA1, CA3 and DG, respectively (Figures 6A,B,D; Table 3). In the ventral hippocampus, the percentage of NPY and SST dual-labeled neurons was 52.4, 71.4 and 93.5% in the CA1, CA3, and DG, respectively (Figures 6A,C,E; Table 1). Interestingly, we found that NPY also co-localized with PV expressing interneurons, in particular in the dorsal hippocampus. There, 9.5, 15.6, and 14.9% expressed NPY and PV (Figures 7A,B,D; Table 4), while in the ventral hippocampus the co-localization was only 2.0, 3.7, and 2.2% for CA1, CA3, and DG, respectively (Figures 7A,C,E; Table 4). In order to confirm the co-localization of NPY and PV in the dorsal DG, we performed dual immunohistochemistry in C57Bl6/NCrl wild type mice. Similar to the results in NPY-GFP mice, we found that 15.7% of NPY neurons in the dorsal DG co-express PV (Supplementary Figure 3).

**Table 2:** Co-localization of NPY-GFP and GABA in the hippocampus.

Region	Subdivision	Single-labeled NPY-GFP+ neurons	Single-labeled GABA+ neurons	Double-labeled neurons	Percent of single-labeled NPY-GFP+ neurons	Percent of single-labeled GABA+ neurons	Percent of double-labeled Region neurons
Dorsal HC	CA1	12	229	376	1.9	37.1	60.9
	CA3	2	128	221	0.6	36.5	63.0
	DG	13	31	269	4.2	9.9	85.9
Ventral HC	CA1	16	565	575	1.4	48.9	49.7
	CA3	13	610	315	1.4	65.0	33.6
	DG	3	30	44	3.9	39.0	57.1

*NPY-GABA: n = 3*.

**Table 3:** Co-localization of NPY-GFP and SST in the hippocampus.

Region	Subdivision	Single-labeled NPY-GFP+ neurons	Single-labeled SST+ neurons	Double-labeled neurons	Percent of single-labeled NPY-GFP+ neurons	Percent of single-labeled SST+ neurons	Percent of double-labeled Region neurons
Dorsal HC	CA1	553	154	62	71.9	20.0	8.1
	CA3	278	93	108	58.0	19.4	22.5
	DG	405	43	116	71.8	7.6	20.6
Ventral HC	CA1	558	346	994	29.4	18.2	52.4
	CA3	194	61	637	21.7	6.8	71.4
	DG	1	7	116	0.8	5.6	93.5

*NPY-SST: n = 4*.

**Table 4:** Co-localization of NPY-GFP and PV in the hippocampus.

Region	Subdivision	Single-labeled NPY-GFP+ neurons	Single-labeled PV+ neurons	Double-labeled neurons	Percent of single-labeled NPY-GFP+ neurons	Percent of single-labeled PV+ neurons	Percent of double-labeled Region neurons
Dorsal HC	CA1	358	129	51	66.5	24.0	9.5
	CA3	173	102	51	53.1	31.3	15.6
	DG	330	36	64	76.7	8.4	14.9
Ventral HC	CA1	910	326	25	72.2	25.9	2.0
	CA3	603	127	28	79.6	16.8	3.7
	DG	87	1	2	96.7	1.1	2.2

*NPY-PV: n = 3*.

## Discussion

While the NPY system has demonstrated considerable potential in modulating emotional-affective processes (Kask et al., 2002; Bowers et al., 2012; Holzer et al., 2012; Verma et al., 2012; Comeras et al., 2019), the contribution of NPY neurons in the dorsal hippocampus was still unclear.

Here, we show that activation of inhibitory neurons in the dorsal DG suppresses cued and trace fear recall, while inhibition of DG GABA neurons had no detectable effect. Interestingly, concomitantly released NPY did not contribute to cued or trace fear expression, but rather suppressed the contextual component in a foreground-conditioning paradigm and promoted extinction learning. This finding clearly disentangles effects exerted by the principal neurotransmitter GABA from those of NPY released simultaneously by the same interneuron population in the dorsal DG.

Pavlovian fear conditioning is a form of associative learning that replicates essential aspects of PTSD in animal models (Bowers and Ressler, 2015). Although simple in nature, it has the capacity to investigate the contribution of individual brain areas by various modifications of the original paradigm (Misane et al., 2005; Esclassan et al., 2009; Herry et al., 2010; Daldrup et al., 2015). For instance, the strength of the pairing between the initially neutral CS and the aversive US can be influenced by subtle changes of the separating time interval. Co-termination of CS/US strongly depends on amygdala processing, while increasing the time interval, the so-called “trace”, will eventually involve several other brain areas, such as the hippocampus as well as several cortical areas. Trace fear may further trigger complex learning events, including safety learning and correct timing (Raybuck and Lattal, 2014). While the ventral hippocampus provides general guidance for emotional-affective regulation, including anxiety and fear-related responses, the dorsal hippocampus seems to play a more selective role in trace fear processing (Raybuck and Lattal, 2014). Importantly, we optimized trace fear conditioning for our mouse strains ultimately deciding for a 5 s trace interval. This was a crucial step in order to avoid alternative learning processes, such as explicit un-pairing and safety learning. Thus, we tested the influence of inhibitory neurons of the dorsal DG in trace fear retrieval. We demonstrated that the activation of DG GABA neurons reduced fear expression to the cue and trace, while inhibition of the same neurons did not have an obvious effect. Accordingly, the increasing inhibitory tone may interrupt the recall of the CS-trace-US association, while increasing excitatory drive during trace fear recall would not further enhance memory-dependent freezing behavior. Such a concept could support the conventional hypothesis suggesting that the preservation of neuronal activity bridges the trace period. However, recently this view has been challenged by two studies stating that temporal coding and sequential activity are not underlying trace fear learning. Zhang et al. (2019) reported that in fact specific neuronal ensembles in the CA1 of the dorsal hippocampus were activated during fear retrieval by the CS presentation and by the stimulus-free trace period likewise. In a similar approach, it was proposed that both, CS and trace, were encoded by a sparse subset of hippocampal pyramidal neurons forming a specific engram (Ahmed et al., 2020). Thus, in our study, the activation of DG GABA neurons during fear retrieval may have inhibited the reactivation of a specific fear engram equally encoding for CS-related and trace-related memories. If on the other hand, activation of DG GABA neurons interrupted the temporal bridging of CS and trace, one would expect to see a specific reduction of trace-related freezing but not CS-induced freezing. We, however, found that freezing behavior to the CS and trace was fairly correlated across all treatment groups albeit at different expression levels, supporting a general reduction of specific fear engram cell activation (Figure 1G). There may be several reasons why inhibition of DG GABA neurons did not enhance fear recall. Most likely, a ceiling effect of freezing masked the detection of significantly increased freezing levels. Conversely, if dedicated neuronal ensembles control the expression of CS/trace fear, then inhibition of DG GABA neurons would not further enhance the reactivation of a specific engram. In other words, if a CS/trace assigned engram is completely recalled, inhibiting local GABA neurons cannot further promote it, but would rather result in unspecific events, such as fear generalization. Future studies using different US intensities and training schemes, such as differential fear conditioning paradigms with predictive and non-predictive cues may shed more light upon this issue (Verma et al., 2012).

Interestingly, over-expression of NPY with or without consecutive chemogenetic activation of DG GABA neurons did not further modify CS-related and trace-related freezing behavior during fear recall. These data indicate that freezing behavior induced by discrete stimuli, such as CS and trace, is strongly dependent on acute inhibition/excitation balance by GABA and glutamate release, but less on the effects of a modulating neuropeptide transmitter that acts on much longer time scales. Limitations of the current experiment are the very low freezing levels upon chemogenetic activation of DG interneurons, which may have resulted in a floor effect that precluded the detection of further freezing suppression caused by concomitantly released NPY.

Contextual information provides a dedicated aspect of episodic memories, specifying their temporal and spatial representation. In fear conditioning paradigms, the context may consist of a set of features defining the external conditions under which the pairing of a specific CS with a related US was performed. Under these conditions, the context provides a “background” representation and induces, on its own, only weak fear expression during consecutive testing. On the other hand, in “foreground” contextual conditioning the context representation serves as the only predictor and may thus induce full fear memory retrieval. We found that NPY potentially co-released (Noe et al., 2008; Verma et al., 2015) from activated DG GABA neurons significantly reduced the recall of contextually relevant fear in a “foreground” context fear paradigm. In addition, in the same paradigm extinction of context fear was facilitated, resulting also in a reduced freezing time when tested for extinction recall 24 h later and in the absence of CNO injection. Since no additional saline control was present in these experiments, we do not know whether activation of DG GABA neurons alone is sufficient to reduce contextual fear expression. However, the data suggest that simultaneously released NPY may interfere specifically with the contextual representation of hippocampal fear engrams, while it is not involved in the processing of CS-related or trace-related hippocampal processing. This makes sense when considering context as a complex episodic memory that needs time to be appropriately recollected, as demonstrated by the bell-shaped curve of a typical context fear retrieval session. Thus, slowly developing modulatory activity of neuropeptide transmitters may especially affect this kind of cerebral processing. In contrast, simple and more specific cues, such as a tone and the adjacent trace period, are less susceptible to neuromodulators, but rather depend on the balance of the fast-acting neurotransmitters GABA and glutamate. Therefore, increasing GABAergic tone upon fear recall may affect cued, trace and context fear likewise, while possible co-release of NPY would address specifically the contextual components. While we did not specifically address the question of how NPY overexpression alone would have affected individual aspects of fear processing, it is important to note that overexpression of NPY alone did not modify fear expression during acquisition or background context testing (Figures 2C,D, 3C). Considering the disease course and inherent properties of PTSD, with fear generalization increasing over time and extending towards various triggers and circumstances, interference with the hippocampal NPY system seems to be a promising treatment strategy.

Several classes of interneurons are populating different regions of the hippocampal DG. These inhibitory neurons are orchestrating the activity of dentate granule cells by restricting their activation and assigning dedicated neuronal ensembles to specific contextual representations. In particular, hilar perforant path-associated (HIPP) cells receive inputs from dentate granule cells and perforant path terminals, while targeting granule cell dendrites in the outer molecular layer. These neurons provide powerful feedback inhibition and curtail the size of the hippocampal fear engram. Importantly, in addition to GABA, they release SST and NPY as modulatory neurotransmitters. Recently, it has been suggested that NPY HIPP cells in the DG especially modulate “background” context fear expression by inhibiting DG granule cells (Raza et al., 2017). Y1 receptors seem to be predominantly involved in this phenomenon. Indeed, we showed that in the dorsal DG more than 70% of SST neurons also express NPY.

However, we also demonstrated that NPY is expressed by several other DG interneuron populations, including PV basket cells. DG PV basket cells are activated by granule cells and provide powerful lateral inhibition (Espinoza et al., 2018). This effect limits the size of the resulting fear engram and enables the simultaneous storage and differentiation of multiple highly similar representations, a phenomenon called pattern separation. Activation of PV cells during fear memory recall may increase the specificity of the memory by providing efficient lateral inhibition and protection from the propagation of PTSD-related phenomena, such as fear generalization. We showed that in the dorsal DG, 64% of the PV neurons also contain NPY. The dentate PV cell population consists of basket cells and axo-axonic cells. Thus, co-expressed NPY may reach postsynaptic Y1 Rs on granule cell dendrites as well as pre-synaptic Y2 Rs on granule cells axons, *via* neuropeptide-specific volume transmission. A general inhibition of dentate granule cells may reduce fear expression and at the same time, result in a downscaling of synaptic activity affecting in particular weakly activated granule cells. This in turn could sharpen the overall contrast of the respective neuronal engrams and reduce fear generalization while promoting fear extinction learning.

In order to shed more light on the role of NPY in hippocampus-dependent fear learning, we activated or inhibited DG GABA neurons in VGAT-cre mice, including SST and PV neurons, which were mostly NPY positive. By addressing both neuronal subpopulations, our approach may have inhibited granule cells on various neuronal specializations and targeted dendritic Y1 and axonal Y2R likewise. It is also interesting to note that mere overexpression of NPY in dentate interneurons did not alter fear expression in a conditioning protocol in which context was used in the background. Under the selected conditions, NPY release may have been too weak, or the respective receptors could have been already saturated, precluding a detectable effect of increased NPY levels. However, when we excited these neurons in the presence of NPY, a reduction was seen in conventional context conditioning during fear recall and extinction. Initially, this may contradict the results reported earlier (Raza et al., 2017), however, several methodological subtleties could explain these differences. Further studies are needed to elucidate the role of NPY and in particular of the individual Y receptors in the different variations of Pavlovian fear conditioning.

Taken together, we demonstrated that NPY is expressed by different populations of GABA neurons within the mouse DG, including not only SST but also PV basket cells or axo-axonic cells. Furthermore, we showed that activation of DG GABA neurons reduced CS and trace fear recall. Importantly, concomitantly released NPY did not interfere with DG-dependent modulation of cued or trace fear, but significantly reduced contextual foreground fear, while promoting extinction learning. Thus, co-release of neuropeptide transmitters may affect specific emotional-affective and cognitive aspects of a fear memory and could provide a promising therapeutic add-on for the treatment of PTSD.

## Data Availability Statement

The original contributions presented in the study are included in the article/Supplementary Material, further inquiries can be directed to the corresponding author.

## Ethics Statement

The animal study was reviewed and approved by the Austrian Ministry of Science.

## Author Contributions

The manuscript was prepared by LC, NH, PM, and RT. LC and NH performed the viral injections and the behavioral testing. PM made the histological analysis. RT designed and supervised the experiments.

## Conflict of Interest

The authors declare that the research was conducted in the absence of any commercial or financial relationships that could be construed as a potential conflict of interest.

## Supplementary Material

SUPPLEMENTARY FIGURE 1 Variation of trace length in trace fear conditioning in male C57Bl6/NCrl mice. **(A)** A 20 s trace length between conditioned stimulus (CS) and unconditioned stimulus presentation resulted in an equal percentage of freezing during CS, trace (T), and inter-trial interval (P). **(B)** In contrast, a 5 s trace interval after CS produced an increased percentage of freezing during the trace compared to medium freezing levels upon CS presentation and lowest freezing levels during the inter-trial interval. **(C)** Analysis of a 20 s trace in 5 s bins revealed increased percentage of freezing only during the first 5 s after the CS, but not in the following time intervals. Data are presented as means ± SEM and were analyzed by one-way ANOVA for repeated measurements, **P* < 0.05, *n* = 12.

SUPPLEMENTARY FIGURE 2 Analysis of the injection sites and affected neuronal subpopulations throughout the hippocampal formation. **(A)** Immunohistochemically labeling of RFP in the hippocampus of an rAAV-hSyn-DIO-hM3DGq-mCherry injected VGAT-Cre mouse. Note that neuronal expression is confined to the dorsal DG, however, a lower number of fibers was also present in other hippocampal subregions, probability representing a restricted number of axonal projections. **(B)** Immunohistochemically labeled neurons for parvalbumin (PV, red) and RFP (green) in the granule cell layer of the dorsal DG (arrows illustrate dual-labeled neurons). **(C)** Immunohistochemically labeled neurons for somatostatin (SST, red) and RFP (green; arrows illustrate dual-labeled neurons). LV: lateral ventricle DG: dentate gyrus, CA1, 2 and 3: cornu ammonis 1, 2, and 3, respectively. Scale bars: **(A)**: 200 μm, **(B,C)**: 50 μm.

SUPPLEMENTARY FIGURE 3 Specific reduction in context fear but not in cued or trace fear following over-expression of NPY in VGAT neurons of the dorsal dentate gyrus (DG). **(A)** rAAV-hSyn-DIO-hM3DGq-mCherry/rAAV-EF1a-DIO-NPY (Gq + NPY) injected mice freeze less than those injected with rAAV-hSyn-DIO-hM3DGq-mCherry (Gq) alone during context fear test. **(B)** There was no difference in freezing time during cued and trace fear test between Gq + NPY and Gq alone injected mice after reconditioning. **(C)** There was no correlation between the freezing levels to CS and context fear. Both Gq + NPY and Gq alone mice were injected 30 min before the test with CNO (ip, 1 mg/kg). Data are presented as means ± SEM, **P* < 0.05, Gq: *n* = 4, Gq + NPY: *n* = 5.

SUPPLEMENTARY FIGURE 4 Immunohistochemical distribution and quantification of co-expression of parvalbumin (PV) with NPY in the dorsal dentate gyrus (DG). **(A)** Immunohistochemical labeling for NPY (green) and PV (red) depicting dual-labeled neurons (upper panel: individual immunoreactivities in the dorsal dentate gyrus, lower panel: dual-labeling and higher magnification) in male C57Bl6/NCrl mice. **(B)** Histogram depicting the percentage of NPY, PV, and dual-labeled neurons in the dorsal DG. **(C)** Tabular results of respective quantifications. Scale bars: lower magnification 200 μm, higher magnification 50 μm. Abbreviations: MoDG, molecular layer of the dentate gyrus; GCL, granule cell layer; PoDG, hilus/polymorph layer of the dentate gyrus; SGZ, subgranular zone.
