# Supplementary figures and images for: NPY Released From GABA Neurons of the Dentate Gyrus Specially Reduces Contextual Fear Without Affecting Cued or Trace Fear
Source: Front Synaptic Neurosci. 2021 May 26;13:635726. doi: 10.3389/fnsyn.2021.635726 (PMC8187774; doi:10.3389/fnsyn.2021.635726)

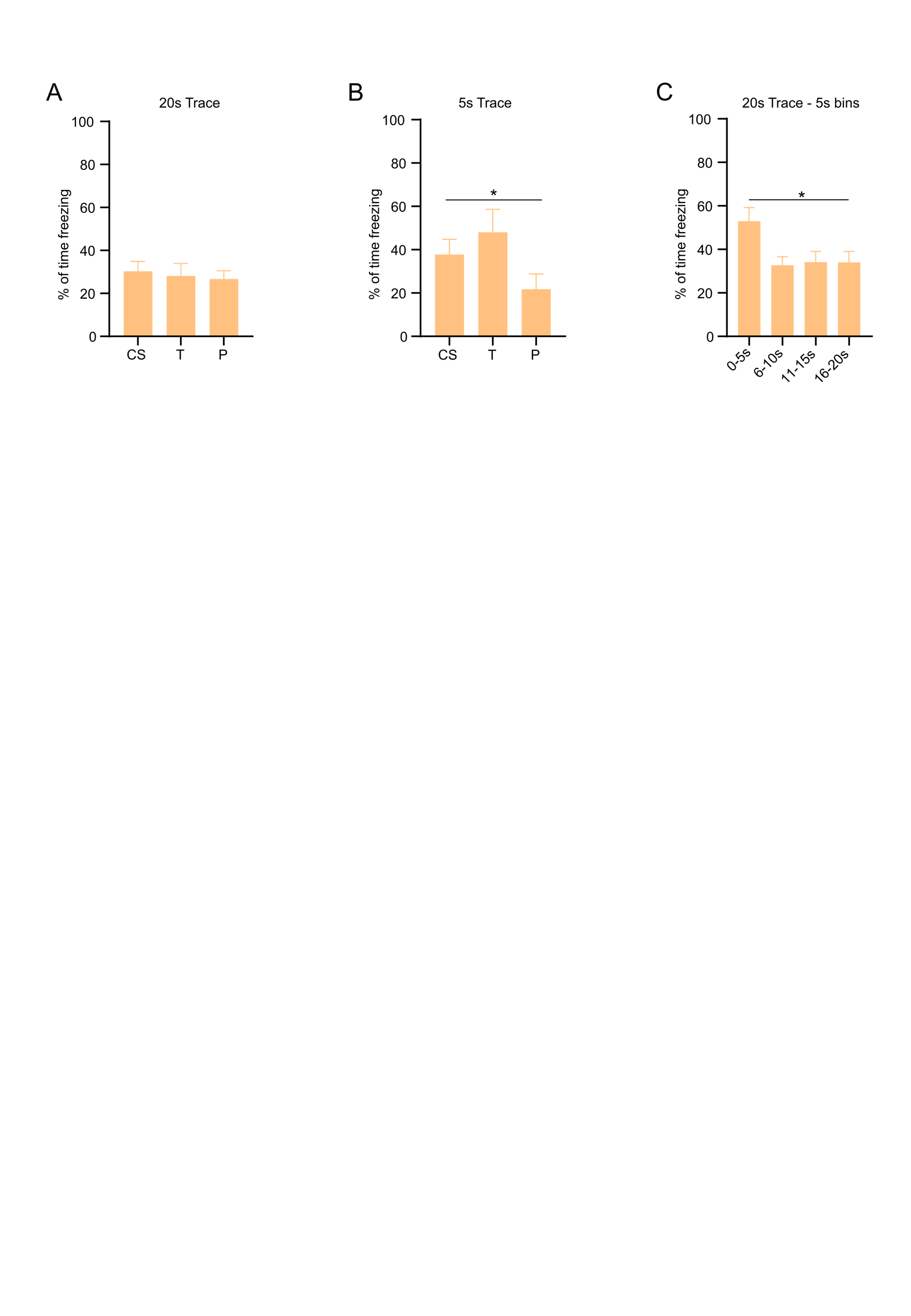

Supplement: SUPPLEMENTARY FIGURE 1 — Variation of trace length in trace fear conditioning in male C57Bl6/NCrl mice. (A) A 20 s trace length between conditioned stimulus (CS) and unconditioned stimulus presentation resulted in an equal percentage of freezing during CS, trace (T), and inter-trial interval (P). (B) In contrast, a 5 s trace interval after CS produced an increased percentage of freezing during the trace compared to medium freezing levels upon CS presentation and lowest freezing levels during the inter-trial interval. (C) Analysis of a 20 s trace in 5 s bins revealed increased percentage of freezing only during the first 5 s after the CS, but not in the following time intervals. Data are presented as means ± SEM and were analyzed by one-way ANOVA for repeated measurements, *P < 0.05, n = 12. [file Image_1.TIF]

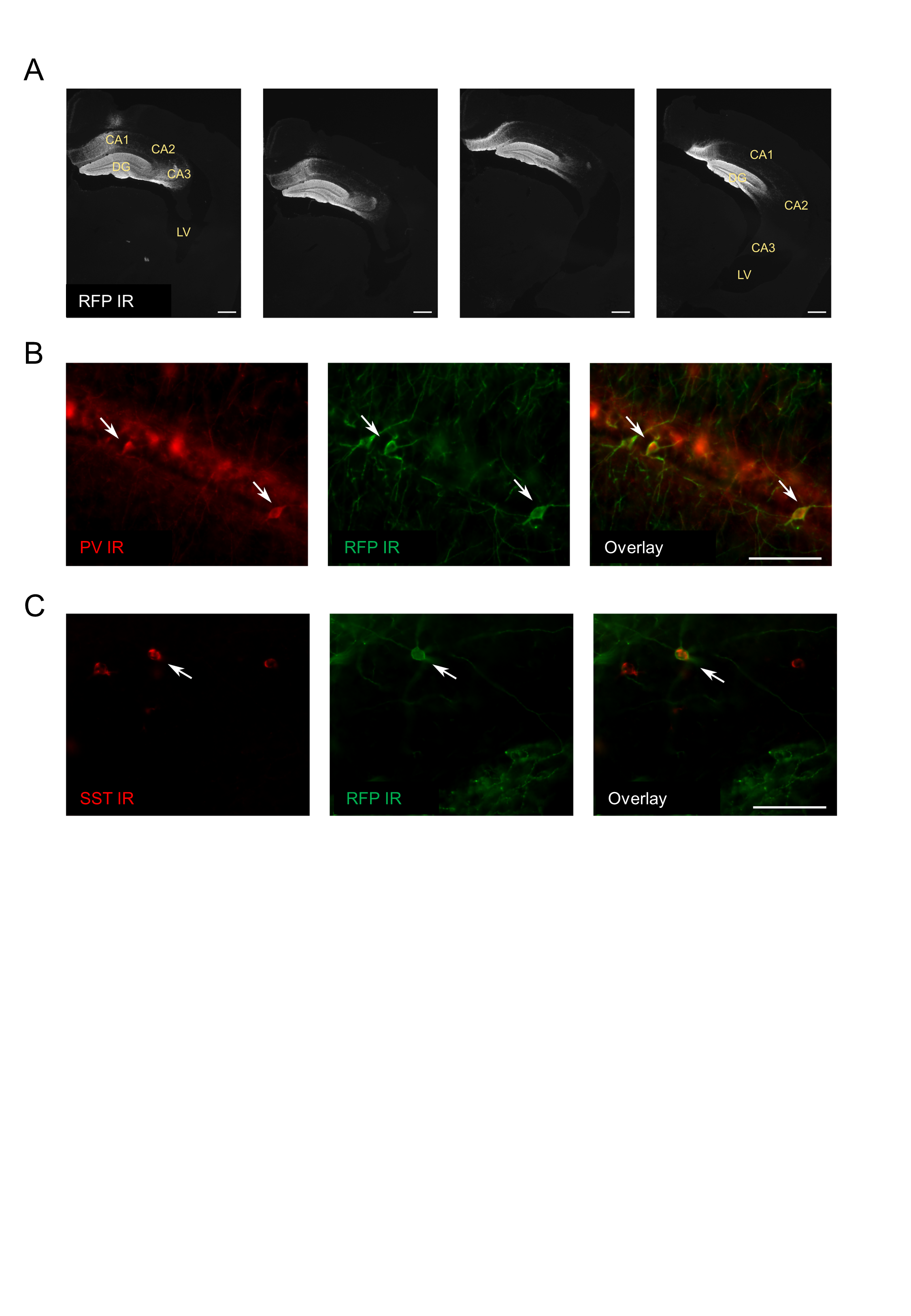

Supplement: SUPPLEMENTARY FIGURE 2 — Analysis of the injection sites and affected neuronal subpopulations throughout the hippocampal formation. (A) Immunohistochemically labeling of RFP in the hippocampus of an rAAV-hSyn-DIO-hM3DGq-mCherry injected VGAT-Cre mouse. Note that neuronal expression is confined to the dorsal DG, however, a lower number of fibers was also present in other hippocampal subregions, probability representing a restricted number of axonal projections. (B) Immunohistochemically labeled neurons for parvalbumin (PV, red) and RFP (green) in the granule cell layer of the dorsal DG (arrows illustrate dual-labeled neurons). (C) Immunohistochemically labeled neurons for somatostatin (SST, red) and RFP (green; arrows illustrate dual-labeled neurons). LV: lateral ventricle DG: dentate gyrus, CA1, 2 and 3: cornu ammonis 1, 2, and 3, respectively. Scale bars: (A): 200 μm, (B,C): 50 μm. [file Image_2.TIF]

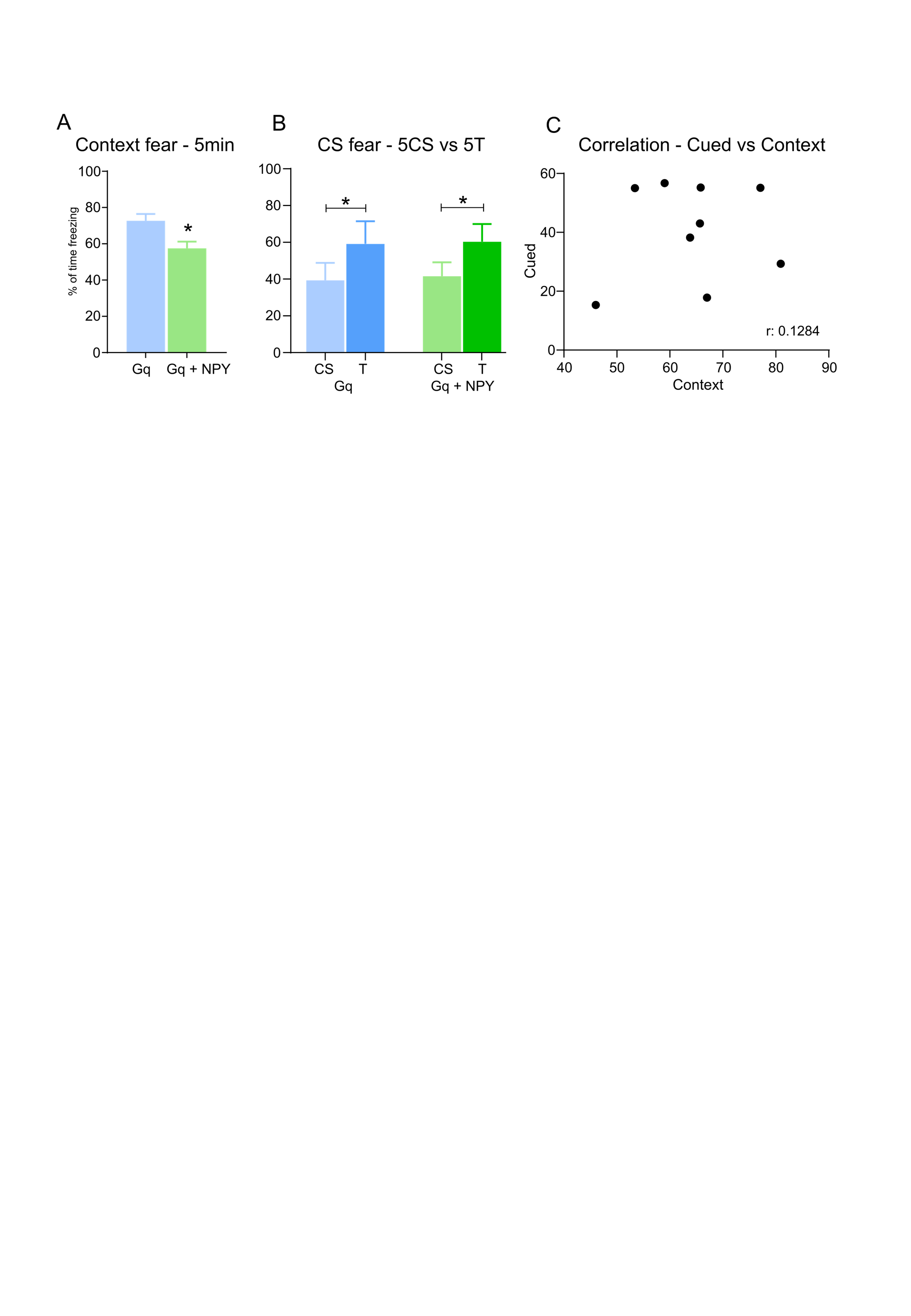

Supplement: SUPPLEMENTARY FIGURE 3 — Specific reduction in context fear but not in cued or trace fear following over-expression of NPY in VGAT neurons of the dorsal dentate gyrus (DG). (A) rAAV-hSyn-DIO-hM3DGq-mCherry/rAAV-EF1a-DIO-NPY (Gq + NPY) injected mice freeze less than those injected with rAAV-hSyn-DIO-hM3DGq-mCherry (Gq) alone during context fear test. (B) There was no difference in freezing time during cued and trace fear test between Gq + NPY and Gq alone injected mice after reconditioning. (C) There was no correlation between the freezing levels to CS and context fear. Both Gq + NPY and Gq alone mice were injected 30 min before the test with CNO (ip, 1 mg/kg). Data are presented as means ± SEM, *P < 0.05, Gq: n = 4, Gq + NPY: n = 5. [file Image_3.TIF]

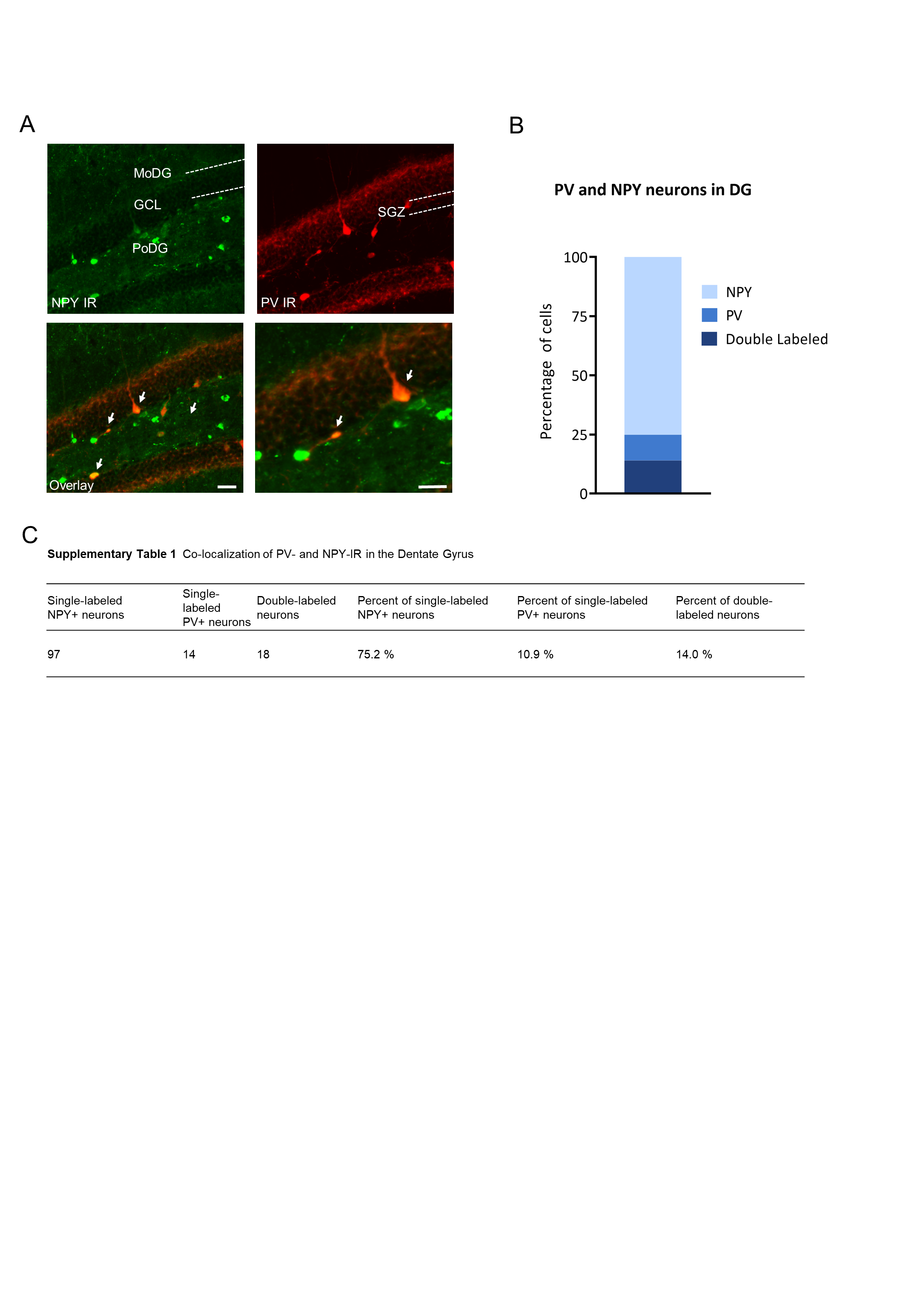

Supplement: SUPPLEMENTARY FIGURE 4 — Immunohistochemical distribution and quantification of co-expression of parvalbumin (PV) with NPY in the dorsal dentate gyrus (DG). (A) Immunohistochemical labeling for NPY (green) and PV (red) depicting dual-labeled neurons (upper panel: individual immunoreactivities in the dorsal dentate gyrus, lower panel: dual-labeling and higher magnification) in male C57Bl6/NCrl mice. (B) Histogram depicting the percentage of NPY, PV, and dual-labeled neurons in the dorsal DG. (C) Tabular results of respective quantifications. Scale bars: lower magnification 200 μm, higher magnification 50 μm. Abbreviations: MoDG, molecular layer of the dentate gyrus; GCL, granule cell layer; PoDG, hilus/polymorph layer of the dentate gyrus; SGZ, subgranular zone. [file Image_4.TIF]
